# Cognition, Emotion, and Movement in the Context of Rehabilitation

**DOI:** 10.3390/ijerph192114532

**Published:** 2022-11-05

**Authors:** Tal Krasovsky

**Affiliations:** 1 Department of Physical Therapy, University of Haifa, Haifa 3498838, Israel; tkrasovsk@univ.haifa.ac.il; 2 Pediatric Rehabilitation Department, The Edmond and Lily Safra Children’s Hospital, Sheba Medical Center, Ramat-Gan 52621, Israel

This Special Issue aims to advance the state of inquiry into the interaction between emotions, cognition, and motor performance and learning. Motor performance and emotions have a bidirectional relationship, demonstrating that, on the one hand, an altered emotional state is associated with a change in motor performance [1] and on the other hand, changes in movement production (e.g., intensity, duration) lead to a cascade of physiological (e.g., metabolic, hormonal) processes that impact mood, including symptoms of depression and anxiety [2]. The goal of rehabilitation of motor function following neurological or orthopedic impairment is to promote motor learning, defined as the relatively permanent changes in motor performance in movement capability [3]. Emotions may play an important role in motor skill acquisition. Wulf & Lewthwaite [4] constructed a framework for performance optimization based on three pillars (referred to as the OPTIMAL theory of motor learning). The first pillar is enhanced expectancies: “purposeful manipulations within training environments that safely enhance a learner’s confidence for achieving success in a prescribed task” [4], p. 18. Thus, manipulations aimed to enhance the learner’s confidence may improve their learning in a rehabilitation context as well. Indeed, positive affect at discharge from hospital is an independent predictor of both cognitive and motor function following a stroke [5]. Furthermore, the generation of positive affect is an inherent motivation for using novel interventions in motor rehabilitation. For example, a primary rationale for using virtual reality as a rehabilitation intervention is its potential to promote positive affect, leading to increased motivation and engagement and potentially to improved motor learning [6]. However, the use of affective terminology in rehabilitation research is at times inaccurate, and measurement of potential interactions between affective states, cognitive and motor performance and learning needs to be further emphasized [6].

Specific characteristics of emotions are known to differentially affect motor function. Experienced and expressed emotions can qualitatively be characterized using a bipolar dimensional categorization of valence (i.e., pleasant or unpleasant) and arousal (i.e., intensity level) [7]. Using this categorization, arousal is a key factor in triggering physiological responses (e.g., increased heart rate, sweating) and preparing the motor system for action (reducing reaction times and increasing force production) [1]. For upper limb motor tasks, unpleasant stimuli provoke a faster and stronger motor response compared with valence-matched pleasant stimuli [8,9]. For standing balance, unpleasant stimuli (postural threat) are known to alter the amplitude and spectral characteristics of postural sway (for a review, see [10]. Finally, the spatiotemporal characteristics of gait, ranging from gait speed to smoothness, are altered by emotions in a valence-dependent manner [11,12], and these alterations serve as points of departure for the emotion detection of walkers by human observers [13,14] and machine learning algorithms [15]. Emotions alter balance and gait not only as a state but also as a trait. Thus, people with a tendency for higher conscious movement processing lean backwards to a greater extent during a static balance task with a postural threat [16] and people with diagnosed depression [17] and anxiety [18] walk with a decreased gait speed.

When responding to everyday experiences, people tend to use different emotion regulation strategies, some of which have been demonstrated to be maladaptive and associated with psychopathology such as anxiety and depression [19]. Importantly, emotional regulation strategies vary in their effectiveness to produce changes in motor performance [20]. For example, suppressing emotional expression, which is a strategy for emotional regulation, is associated with slower action planning in young adults [21], and with slower dual-task walking in older adults [22], suggesting that in addition to emotional state, emotional regulation strategies interfere with the executive functioning required for performance of motor tasks. These results, together with work demonstrating increased activation of brain areas associated with attention when processing emotional visual stimuli [23,24], further highlight the importance of attentional focus in the regulation of emotions accompanying motor performance.

Despite the compelling evidence linking emotions to motor performance, and while the importance of emotions and emotional regulation has been extensively emphasized in other fields, such as education [25] and exercise sciences [1], less is understood about the relationship between emotions and cognitive and motor performance in a rehabilitation context. The papers in this Special Issue will align with a recent movement towards accounting for the role of emotional factors in rehabilitation effectiveness. Recently, Beatty and Janelle [1] advocated for the integration of emotional regulation strategy considerations in the context of optimizing motor performance in sports. Other researchers (e.g., [26] encourage the integration of affective states into models of motor learning. Papers in this Special Issue will contribute to this discussion and identify new areas for research to better understand essential relationships between emotions, cognition, and motor performance and learning in the context of rehabilitation.
